# The Relationship between Cyberbullying and traditional Bullying

**DOI:** 10.1192/j.eurpsy.2023.1529

**Published:** 2023-07-19

**Authors:** L. Sahli, S. Bourgou, S. Haj Amor, A. Belhadj

**Affiliations:** 1 child and adolescent Psychiatry, CHU Mongi Slim, Tunis, France; 2 child and adolescent Psychiatry, CHU Mongi Slim; 3 Regional Directorate of Tunis Carthage Health District, Tunis, Tunisia

## Abstract

**Introduction:**

Traditional bullying and cyberbullying behaviors represent a serious problem in our schools with deterious effects on youths.

**Objectives:**

The aim of our study is to determine the relationship between school bullying and cyberbullying among tunisian adolescents.

**Methods:**

Adolescents enrolled in middle and high schools in Tunis, Tunisia were surveyed about their experiences of traditional bullying and cyberbullying. This study was developed by the Child Psychiatry Department of Mongi Slim Hospital, (Tunis,Tunisia). Approval of the ethic commitee of the Hospital was obtained.

**Results:**

The total number of participants in our study was 935 adolescents. The average age was 14.2 years with a slight female predominance (54%) and a sex-ratio of 0.85.

The results revealed that 32 % of the students were victims of both cyber and traditional bullying, while 26 % of the students bullied others in both cyber and physical environments. Compared to female students, male students were more likely to be bullies and victims in both physical and cyber-environments.

**Conclusions:**

Cyberbullying and traditional bullying may not be two separate phenomena, but rather two sides of the same coin. Reducing bullying is an important issue to deal with, wheatear it happens online or offline.

**Disclosure of Interest:**

None Declared

